# A Null Relationship between Media Multitasking and Well-Being

**DOI:** 10.1371/journal.pone.0064508

**Published:** 2013-05-15

**Authors:** Shui-I Shih

**Affiliations:** Psychology, University of Southampton, Southampton, United Kingdom; The University of Queensland, Australia

## Abstract

There is a rapidly increasing trend in media-media multitasking or MMM (using two or more media concurrently). In a recent conference, scholars from diverse disciplines expressed concerns that indulgence in MMM may compromise well-being and/or cognitive abilities. However, research on MMM's impacts is too sparse to inform the general public and policy makers whether MMM should be encouraged, managed, or minimized. The primary purpose of the present study was to develop an innovative computerized instrument – the Survey of the Previous Day (SPD) – to quantify MMM as well as media-nonmedia and nonmedia-nonmedia multitasking and sole-tasking. The secondary purpose was to examine whether these indices could predict a sample of well-being related, psychosocial measures. In the SPD, participants first recalled (typed) what they did during each hour of the previous day. In later parts of the SPD, participants analysed activities and their timing and duration for each hour of the previous day, while relevant recall was on display. Participants also completed the Media Use Questionnaire. The results showed non-significant relationship between tasking measures and well-being related measures. Given how little is known about the associations between MMM and well-being, the null results may offer some general reassurance to those who are apprehensive about negative impacts of MMM.

## Introduction

Media exposure has rapidly risen over recent years and, with it, the propensity for media-media multitasking (MMM) – the concurrent use of two or more media (e.g., phone, email, iPod). Between 1999 and 2009, the average media use reported by Americans aged between 8 and 18 years grew from 6.3 to 7.6 hours a day, of which MMM increased from 16% to 29% [1]. For young people in the UK, average reported media use in 2010 for 12 to 15 and 16 to 24 year olds was 6.3 and 9.5 hours respectively, of which 15% and 52% involved MMM [2]. The extent of media exposure is positively related to risk-taking and sensation-seeking behaviours and negatively related to personal adjustment and school performance [3], [4]. However, implications of MMM may go beyond increased media exposure and its related impacts. It has been speculated that MMM may promote shallow engagement, impulsivity, and poor use in language (e.g., arbitrary abbreviations, lack of serious editing) because MMM often involves computers and text communications (e.g., emailing, social networking, texting) that feed frequent interruptions (e.g., new alerts) and encourage prompt responses [2], [3], [5]. The inherent multitasking nature of using social networking services may have contributed to a decline in empathy due to trade-off between virtual and face-to-face contacts [6].

Multitasking involves complex processes within working memory (WM) in order to, for example, manage and update goals, prioritise relevant information, suppress inappropriate actions, and allocate attention. For decades, behavioural and brain research suggests that multitasking is challenging and often stressful and unproductive. However, most multitasking research observes participants conducting two cognitive tasks that do not involve media per se (except that tasks are often computerised) [7]. More importantly, there may be a fundamental difference between multitasking that is externally imposed (for example, at work places or in laboratory experiments) and MMM which is self-indulgent (for example, a need to remain “connected,” to fight off boredom, to fill the waiting time during social networking, or to regulate the mood using music of a particular genre). Hence, research findings on multitasking in laboratory tasks may not be directly applicable to MMM.

The rapidly increasing trend in MMM and the lack of directly applicable research prompted concerned US scholars from diverse disciplines and professionals from education, business, and advocacy to assemble at Stanford University in July 2009 to begin to consider multidisciplinary research to investigate its current and potential impacts [7]. Their concern was that indulgence in MMM may compromise well-being and/or cognitive abilities [8]. It is clear from the Stanford conference that research on MMM is urgently needed in order to improve general understanding as to whether MMM should be encouraged, managed, or minimized (assuming that it is possible to influence usage). The present study helps to fill that gap of knowledge in two ways. First, it developed the Survey of the Previous Day to estimate the extent of not only MMM but also media-nonmedia multitasking (MNM), nonmedia-nonmedia multitasking (NNM) and sole-tasking (ST). It is important to consider different forms of tasking at the same time in order to understand, for example, whether multitasking is a general habit or whether there is a trade-off among them. Second, it explored whether different forms of tasking were associated with a selection of well-being related measures.

A crucial consideration is how to measure or quantify multitasking. The Kaiser Family Foundation (which provides the largest and most comprehensive data about media use among American youth) used Media Use Diaries (MUD) in a series of large-scale studies in the USA [1]. Participants were asked to complete the diaries over a 7-day period (6 am to 12 am each day). The diary was divided into 30-minute timeslots. For each timeslot (e.g., 8:00–8:30 pm), participants first indicated whether they were doing any media activities for at least 15 minutes. If they were, they would be asked to circle their main media activity (out of 12 listed activities) and then to indicate what else they were doing. Thus, playing a video game for 12 minutes would not be counted irrespective if there were any other activities involved; listening to music for 20 minutes with 10 minutes at the end of one timeslot and the remaining time in the next timeslot would not be counted either. Furthermore, when two or more activities being identified for a given timeslot, it is unclear the extent to which they were carried out at the same time. For example, one may have played a game for 10 minutes, stopped playing to answer a call for 5 minutes, then back to the game for the remaining 15 minutes. The timeslot in this example would be credited for multitasking although no two activities were simultaneously carried out. The issue of concurrency is addressed by the Media Use Questionnaire or MUQ [9], which consists of two parts. In the first part, participants estimate the number of hours per week that they normally spend on each of 12 media (e.g., television, non-music audio, email). In the second part, participants indicate, when using each of the 12 media as the primary activity, how often they concurrently use each of the remaining media. Greenberg et al. [10] demonstrated that survey methods (e.g., self-report of the number of TV time in a day or week as in MUQ) were less accurate than diary methods (e.g., log media use activities throughout the course of one particular day). Without clear instructions regarding how to estimate, it is unclear how participants estimate these hours in the MUQ. In summary, both MUD and MUQ focus on media use, disregard other types of tasking (e.g., nonmedia-nonmedia multitasking), and have room for improvement (e.g., to minimise omissions, to provide objective methods for estimation).

The primary aim of the present study was to develop a new instrument – the Survey of the Previous Day (SPD) – that can capture different forms of multitasking as well as sole-tasking, minimize omissions (e.g., not counting activities engaged for less than 15 minutes), lessen the burden on participants (i.e., remember to complete diary as instructed for seven days), offer an objective method of estimation, and hence increase reliability of measures. In the SPD participants wrote/typed what they did during each hour of the previous day. Next, hour by hour the descriptions were fed back to them (i.e., as memory aide) to identify activities (media or nonmedia) engaged in each hour and to indicate during which of the six 10-minute timeslots of the hour each activity had occurred. Thus, an identified activity would be counted no matter how briefly it was engaged in. For any two activities appearing in the same timeslot(s), they then rated the extent to which these were carried out concurrently in the given hour. Times spent on MMM, MNM, NNM, and ST were respectively estimated from the SPD to index the extent of different forms of tasking for each individual. Hence, the SPD is similar to diary methods in that it requires participants to describe and then analyse activities for each hour of the previous day. In the present study, the SPD was completed during a laboratory session rather than left to participants to decide where, when and how to complete the survey. Further multitasking related measures were obtained from two questionnaires – the MUQ and the Multitasking Preference Inventory [11].

Descriptions written in the SPD serve two functions. First, they provide memory aide for analysing activities and their timing in later parts of the SPD. Second, they can be used to derive psychosocial variables. For more than a decade, Pennebaker and his colleagues have demonstrated that daily word use can reveal psychosocial aspects about individuals [12], [13]. Even function words (e.g., I, me, he, for, of, can) play a crucial role in probing emotions and social skills [14]. In the present study, the descriptions were submitted to Linguistic Inquiry Word Count or LIWC [15] to obtain constituent variables of psychosocial constructs (see Method).

The secondary aim of the present study was to examine whether multitasking was associated with well-being related, psychosocial factors. In addition to variables derived from descriptions in the SPD, a handful of well-validated questionnaires were used to measure variables that have been related to subjective well-being – sensation seeking [16], Big Five traits [17], general capacity to control attention [18], and impulsivity [19]. Regression analyses were used to explore the relationship between tasking indices and respective psychosocial measures.

## Methods

This project was approved by the Ethics Committee of Psychology and the Research Governance Office and Insurance Services, University of Southampton. All participants were treated according to the ethical standards of the British Psychological Society. All participants gave informed consent by typing Y to indicate that they had understood on-screen instructions and that they gave consent to participate. Their responses to consents were stored in electronic data files. This consent procedure was approved by the Ethics Committee.

### Participants and Apparatus

Data obtained from 138 participants (27 males) were analysed. Participants were psychology undergraduates at the University of Southampton (age: 18–43, *M* = 20.6, *SD*  = 3.5). The majority were second year students who took part to partially fulfil the course requirement of a laboratory module. The remaining (*n* = 17) were first year students who participated voluntarily. They all gave consent for their data to be used for further research and publication. Tasks were administered in groups of 6 to 30 participants in a large teaching laboratory. Individual computers controlled and displayed instructions and stimuli. All completed the SPD; 117 of them completed a battery of questionnaires.

### Materials

#### The Survey of the Previous Day (SPD)

This 4-part survey was computerized using Python and Tkinter toolkit [20] to support customized items and options. The MUQ was later added as Part 5 and was thus not completed by 15 participants (out of 138).

#### *Part 1: Describe each hour*

Participants were prompted to type a description for each hour of the previous day beginning from 00∶00 to 23∶59, e.g., “For 14∶00–15∶00 of yesterday, please provide detailed descriptions about what you did, how you feel, and its context (where, who else).” Participants were free to revise them during Part 2 and 3.

#### *Part 2: Identify activities*

Descriptions for each hour were displayed. Participants were to identify the activity or activities that appeared in the descriptions from a list of 25 activities (Table 1). The list was finalized via pilot studies, in which five participants (students in different years and one staff member) completed and commented on the survey. The author was present throughout each session of data collection and there was no indication that the list was not inclusive for the current sample.

**Table 1:** The List of 25 Activities Used in the Survey of the Previous Day.

Variable Name	Item descriptions
Media activity	
Screen	Watch screen media (e.g., TV, DVD, youTube)
Audio	Listen to audio media (e.g., radio, mp3, iTune)
Voice	Use voice-based media (e.g., phone, skype)
Text	Use text-based media (e.g., text message, instant message) excluding social networking services
Game	Use gaming media (e.g., computer games, gaming console, portable device)
pRead	Read or browse print media (e.g., books, magazines)
eRead	Read or browse electronic media (e.g., online news, articles, books; e-book reader such as Kindle)
eWork	Write or edit using computer-based applications (e.g., Word, Excel, PhotoShop)
eShop	Visit online stores (including banks)
eSocial	Use social networking services (e.g., Facebook, Twitter, MySpace)
Nonmedia activity	
pSocial	Interact with people face-to-face (including ordinary and intimate relations)
Class	Attend classes (e.g., lecture, tutorial, lab)
pWork	Work on paper (e.g., artwork, notes, letters, essays, Sudoku)
Employment	Carry out employment duties that do not involve computer-based applications
Care	Provide care for close relations (e.g., children, parents)
Chores	Carry out household chores (e.g., cooking, cleaning)
pShop	Visit physical stores (including banks, markets, surgeries, concert halls, theaters)
Foot	Commute on foot or a bicycle
Car	Commute by private vehicles (including motorbike)
Bus	Commute by public transportation (e.g., bus, train)
Exercise	Physical exercise (indoors or outdoors)
Wait	Wait (to be served or for someone/thing)
Personal	Attend to personal appearance or hygiene
Eat	Eat or drink (including cigarette, alcohol, etc.)
Sleep	Sleep or nap

#### *Part 3: Analyse activities*

For each hour, if more than one activity was identified, they were listed. For each activity, participants indicated when it took place by ticking applicable 10-minute timeslots (a recommended interval for time use survey [21]) regardless how brief it was. Where activities had appeared in overlapping timeslot(s), they were presented pairwise. For each pair, the participants indicated the extent to which they were performed concurrently in that hour – rarely, sometimes, about half the time, frequently, or almost always.

#### *Part 4: General experience with multitasking*

A list of activity pairs identified in Part 3 (excluding those ‘rarely’ performed together) was displayed. For each pair, participants indicated for how long [1 month or less, 1–6 months, 6–12 months, 12–24 months, or more than 24 months] and how frequently [rarely, occasionally, often, or almost always] such pairing had occurred in their life. Data from this part were not included in the present report because suitable algorithms to quantify these ratings are yet to be examined.

#### *Part 5: Media Use Questionnaire or MUD [9]*

Participants first reported the number of hours per week they normally spent on each of the 12 media – print, television, computer-based video, music, non-music audio, video or computer games, web surfing, other computer-based applications, telephone and mobile phone voice calls, instant messaging, SMS (text messaging), and email. For each medium, they then indicated, while using it as the primary activity, how often they concurrently consumed each of the remaining 11 media. A media multitasking index or MMI was computed according to the formula of Ophir et al [9].

#### Language Analyses

Descriptions regarding waking hours from the SPD were submitted to the LIWC [15], which outputs word count and the percentage of words in 80 linguistic (e.g., article, pronoun, verb, negation) and psychosocial (e.g., affective, cognitive, perceptual, and social processes) variables. According to the statistics published in the LIWC manual, mean word count for nearly 3000 “emotional writing” was about 450 words and that for nearly 2500 “control writing” was about 400 words. From the present sample, mean and median word counts were respectively 889 and 722 words, providing sufficient materials for the LIWC analysis. For the present purpose, five scores were derived from the analysis: (total) word count and four composite scores. Word count may be indicative of sociability because it has been associated with talkativeness and verbal fluency [22] and sociability is strongly associated with positive affect and life satisfaction [23]. Composite scores were the averages of standard scores of constituent variables and they were computed as follows:

#### *Emotional positivity*

This score was computed according to Cohn et al. [24]. The score was defined by subtracting the LIWC score for negative emotion words (e.g., afraid, cry, upset) from the LIWC score for positive emotion words (e.g., hope, smile, relax). Higher scores indicate greater emotional positivity and predict better mental health [25].

#### *Psychological distancing*

This score was calculated according to Cohn et al. [24]. Constituent scores include articles, words of more than six letters, first-person singular pronouns, words indicating discrepancy from reality (e.g., could, should, would), and present-tense verbs. Scores for the last three variables were reversed. A high score suggests “an abstract, impersonal, and rational tone” (p. 689).

#### *Making distinctions*

This score was computed according to Pennebaker and King [13]. Constituent scores include tentative words (e.g., guess, perhaps), negations (e.g., no, never), inclusive words (e.g., and, close, with), exclusive words (e.g., but, except, without), and past-tense words. Scores for the last variable were reversed. This factor reflects cognitive complexity; people score high in this factor tend to be more open to new experiences [14].

#### *Social engagement*

High scores on social processes are suggestive of interest in social environment and interaction [26], while high scores on function words are associated with sophisticated social skills [27]. Thus, the constituent scores for social engagement include words related to social processes (e.g., family, girl, he, mate, talk, share, they) and function words (e.g., articles, auxiliary verbs, conjunctions, prepositions, pronouns). Higher scores indicated greater interests and skills in socialising.

#### Questionnaires

They were administered online using iSurvey [28] and comprised four sections – (a) information about the study and a required consent confirmation, (b) year of birth and gender, (c) test items, and (d) further consents and debriefing. Section (c) consisted of 153 items from published questionnaires – Multitasking Preference Inventory [11], Warwick-Edinburgh Mental Well-being Scale [29], 54-item Scales of Psychological Well-being [30], Attention Control Questionnaire [31], Barratt Impulsiveness Scale – Version 11 [32], Agreeableness and Extraversion Scales [33], and four-item Sensation Seeking Scale [34]. They were pseudo-randomly mixed in groups of 5 or 6 items from two or more instruments that used the same rating scales. The order of groups and items within a group were independently randomized for each participant. For each scale, high scores indicated high self-ratings on the assessed dimension.

## Procedure

For participants who took part in both research sessions, the sequence and timing of sessions were dictated by the syllabus of the laboratory module and they were as follows. Most completed the questionnaires (about 30 minutes) in the first week of Semester 2 (six completed them in the second week). About half of them completed the SPD (a weekday, mostly Tuesdays; about 60 minutes) in the second week and most of the remaining participants completed it in the third week (four completed it in the fourth week).

## Results

### The Survey of the Previous Day

Table 2 shows the proportion of participants, total time, sole-tasking time, total multitasking time (MT), and proportion of MT for each activity. Computations for the last four variables are outlined in the Appendix S1. As expected, the most time consuming activity was sleep/nap, which amounted to about 8.5 hours, and was comparable to the UK average of 527 minutes (or 8.8 hours) reported for this age group [2]. Next came ‘interact with people face-to-face’ or pSocial (more than 5 hours). Popular media included screen and audio. pSocial had the highest amount of MT (more than 3 hours). Average media use was about 7 hours a day, of which 53% involved multitasking, consistent with a recent report [2]. Excluding sleep/nap, average nonmedia activity time was about 14 hours, of which 59% involved multitasking. Thus, multitasking virtually increased a day from 24 to more than 29 hours (i.e., 8.5+7+14). Across activities, mean (*SE*) proportion of waking time (in actual time, not inflated by multitasking) that spent on single (or solus), two, and more than two activities were respectively 54 (1.6), 32 (1.0), and 14 (1.2)%.

**Table 2:** Total Time, Sole-tasking Time, Multitasking Time, Proportion of Multitasking Time, and Proportion of Participants (P%) for Each Activity in the Previous Day.

	Time in hours								
Variable name	Total		Sole-tasking		Multitasking		Multitasking%		P %
	*Mean*	*SE*	*Mean*	*SE*	*Mean*	*SE*	*Mean*	*SE*	(*n* = 138)
Media activity									
Screen	1.74	0.13	0.88	0.08	0.86	0.09	51	3.0	91
Audio	1.24	0.13	0.20	0.03	1.04	0.12	78	3.0	70
Voice	0.40	0.05	0.29	0.04	0.11	0.02	39	4.1	64
Text	0.97	0.10	0.32	0.04	0.65	0.08	62	3.2	80
Game	0.24	0.05	0.08	0.02	0.16	0.04	64	6.5	25
pRead	0.72	0.09	0.29	0.05	0.43	0.07	62	4.7	52
eRead	0.45	0.05	0.13	0.02	0.32	0.05	63	4.4	59
eWork	0.50	0.06	0.17	0.03	0.33	0.05	62	4.8	54
eShop	0.08	0.02	0.04	0.01	0.04	0.01	54	8.8	20
eSocial	0.73	0.08	0.23	0.03	0.49	0.08	61	3.6	79
Nonmedia activity									
pSocial	5.05	0.20	1.80	0.11	3.25	0.17	62	1.9	98
Class	1.65	0.09	0.86	0.08	0.79	0.07	50	3.7	87
pWork	1.07	0.10	0.24	0.05	0.83	0.09	78	3.4	70
Employment	0.31	0.08	0.10	0.04	0.21	0.06	59	8.7	19
Care	0.30	0.09	0.09	0.03	0.21	0.07	60	8.7	14
Chores	0.63	0.05	0.33	0.04	0.30	0.03	52	3.5	83
pShop	0.27	0.04	0.09	0.02	0.18	0.03	67	5.6	45
Foot	0.79	0.06	0.44	0.04	0.36	0.04	44	3.4	78
Car	0.30	0.06	0.14	0.03	0.17	0.05	54	5.6	39
Bus	0.21	0.04	0.12	0.03	0.09	0.02	48	6.2	30
Exercise	0.42	0.06	0.13	0.03	0.29	0.05	65	5.4	41
Wait	0.40	0.05	0.12	0.03	0.28	0.04	70	4.0	60
Personal	1.15	0.06	0.89	0.05	0.26	0.03	21	2.4	99
Eat	1.70	0.07	0.56	0.05	1.14	0.07	66	2.6	100
Sleep	8.55	0.14	8.48	0.14	0.07	0.02	1	0.2	100

Variable names are defined in Table 1.

*Note*. MT% for each activity only includes the data of participants whose total time for that activity was greater than zero.

#### Activity pairing

For each possible pairing of the 25 activities, two measures were calculated – the total time (in minutes) of such pairing across all participants and the number of participants for whom such pairing was present (see Table 3). The proportion of MT for each activity regarding media, pSocial and nonmedia (excluding pSocial and sleep/nap) activities are displayed in Table 4. [pSocial was isolated from nonmedia activities in Table 4 because of its prevalence shown in Table 3.] Table 3 shows that across participants there were four activities – listen to audio media, pSocial, wait, and eat/drink – that were paired with every other activity (except sleep/nap). Notably, pSocial was the most prevalent activity involving multitasking, which took up 25% of MT across participants, followed by eat/drink (9%) and ‘listen to audio media’ (8%). Table 3 and 4 show that media activities were more likely to couple with media than pSocial or nonmedia activities (42 vs. 23 and 35%), pSocial was more likely to be coupled with nonmedia than media activities (69 vs. 31%), and nonmedia activities were more likely to multitask with pSocial than media and nonmedia activities (43 vs. 30 and 26%). Furthermore, Table 3 (upper left corner) reveals that a large proportion of MMM involves “distant” social interactions (i.e., Text and eSocial). For example, 70% of eSocial time was shared with other media activities; among the MMM time involving screen media (1680 minutes), about 36% was associate with Text and about 24% was associated with eSocial.

**Table 3:** Total Time in Minutes across Participants (in Italics) and Number of Participants (in Bold) for Each Pair of Activities.

		1	2	3	4	5	6	7	8	9	10	11	12	13	14	15	16	17	18	19	20	21	22	23	24	25
1	Screen		*157*	*125*	*604*	*144*	*44*	*93*	*90*	*12*	*411*	*3281*	*247*	*138*	*6*	*106*	*160*	*25*	*10*	*0*	*0*	*0*	*160*	*97*	*1158*	33
2	Audio	**23**		*48*	*446*	*186*	*252*	*258*	*144*	*77*	*878*	*1967*	*25*	*472*	*63*	*56*	*428*	*49*	*386*	*385*	*91*	*438*	*164*	*782*	*735*	144
3	Voice	**9**	**9**		*45*	*101*	*0*	*14*	*9*	*0*	*48*	*99*	*14*	*18*	*0*	*72*	*2*	*7*	*151*	*3*	*34*	*9*	*19*	*54*	*55*	0
4	Text	**39**	**38**	**10**		*140*	*79*	*263*	*66*	*14*	*760*	*1153*	*430*	*148*	*0*	*68*	*62*	*58*	*153*	*15*	*112*	*57*	*94*	*173*	*355*	78
5	Game	**8**	**10**	**2**	**6**		*0*	*48*	*2*	*0*	*116*	*437*	*4*	*21*	*0*	*15*	*2*	*0*	*0*	*0*	*5*	*0*	*43*	*0*	*25*	0
6	pRead	**6**	**11**	**0**	**15**	**0**		*206*	*564*	*0*	*42*	*178*	*261*	*1708*	*0*	*45*	*1*	*0*	*12*	*0*	*32*	*0*	*6*	*15*	*98*	0
7	eRead	**11**	**18**	**4**	**22**	**4**	**14**		*392*	*19*	*567*	*230*	*75*	*355*	*3*	*0*	*10*	*0*	*0*	*0*	*0*	*0*	*38*	*15*	*71*	6
8	eWork	**5**	**8**	**2**	**12**	**1**	**19**	**20**		*3*	*81*	*462*	*208*	*626*	*8*	*2*	*0*	*0*	*0*	*0*	*0*	*0*	*3*	*0*	*66*	2
9	eShop	**1**	**7**	**0**	**2**	**0**	**0**	**4**	**2**		*101*	*97*	*0*	*2*	*6*	*0*	*0*	*0*	*0*	*0*	*0*	*0*	*1*	*0*	*32*	0
10	eSocial	**31**	**37**	**10**	**45**	**6**	**10**	**29**	**15**	**6**		*414*	*83*	*106*	*2*	*28*	*23*	*5*	*31*	*1*	*31*	*0*	*30*	*57*	*250*	24
11	pSocial	**71**	**46**	**21**	**61**	**18**	**15**	**20**	**26**	**7**	**42**		*2283*	*554*	*1448*	*889*	*1198*	*846*	*1621*	*769*	*304*	*1438*	*1106*	*634*	*5441*	42
12	Class	**11**	**6**	**2**	**35**	**2**	**8**	**5**	**8**	**0**	**10**	**79**		*2553*	*0*	*0*	*0*	*0*	*89*	*9*	*21*	*29*	*32*	*7*	*126*	41
13	pWork	**11**	**19**	**3**	**24**	**3**	**35**	**22**	**25**	**2**	**17**	**40**	**45**		*5*	*10*	*9*	*0*	*16*	*0*	*3*	*0*	*28*	*9*	*80*	5
14	Employment	**1**	**3**	**0**	**0**	**0**	**0**	**1**	**1**	**1**	**1**	**17**	**0**	**1**		*4*	*57*	*18*	*4*	*4*	*5*	*75*	*37*	*8*	*23*	0
15	Care	**7**	**3**	**4**	**4**	**2**	**3**	**0**	**1**	**0**	**5**	**13**	**0**	**1**	**1**		*95*	*59*	*9*	*35*	*0*	*27*	*39*	*39*	*92*	69
16	Chores	**22**	**21**	**1**	**15**	**1**	**1**	**2**	**0**	**0**	**9**	**62**	**0**	**1**	**1**	**7**		*6*	*1*	*7*	*0*	*33*	*78*	*91*	*256*	0
17	pShop	**2**	**6**	**3**	**13**	**0**	**0**	**0**	**0**	**0**	**1**	**39**	**0**	**0**	**1**	**2**	**1**		*135*	*5*	*0*	*16*	*174*	*5*	*76*	0
18	Foot	**1**	**16**	**18**	**25**	**0**	**4**	**0**	**0**	**0**	**5**	**67**	**14**	**6**	**1**	**1**	**1**	**15**		*0*	*24*	*151*	*26*	*23*	*103*	0
19	Car	**0**	**14**	**1**	**7**	**0**	**0**	**0**	**0**	**0**	**1**	**32**	**1**	**0**	**1**	**4**	**1**	**1**	**0**		*0*	*7*	*10*	*1*	*42*	99
20	Bus	**0**	**6**	**4**	**11**	**1**	**2**	**0**	**0**	**0**	**4**	**24**	**3**	**1**	**1**	**0**	**0**	**0**	**3**	**0**		*0*	*38*	*9*	*34*	20
21	Exercise	**0**	**12**	**3**	**6**	**0**	**0**	**0**	**0**	**0**	**0**	**37**	**4**	**0**	**2**	**1**	**2**	**1**	**12**	**1**	**0**		*4*	*2*	*75*	0
22	Wait	**9**	**17**	**6**	**21**	**5**	**1**	**6**	**2**	**1**	**6**	**56**	**9**	**5**	**4**	**3**	**8**	**19**	**9**	**2**	**6**	**2**		*34*	*123*	36
23	Personal	**13**	**30**	**7**	**28**	**0**	**2**	**3**	**0**	**0**	**14**	**44**	**1**	**2**	**1**	**5**	**12**	**1**	**2**	**1**	**1**	**2**	**4**		*127*	10
24	Eat	**64**	**37**	**10**	**41**	**6**	**15**	**15**	**12**	**4**	**35**	**116**	**19**	**15**	**4**	**8**	**38**	**9**	**18**	**5**	**3**	**7**	**22**	**24**		0
25	Sleep	**2**	**6**	**0**	**5**	**0**	**0**	**1**	**1**	**0**	**1**	**5**	**3**	**1**	**0**	**4**	**0**	**0**	**0**	**1**	**1**	**0**	**1**	**3**	**0**	

Variable names are defined in Table 1.

**Table 4:** Total Multitasking Time Summed Across Participants and the Proportion of Each Type of Shared Activities for Each Activity.

		% Time in shared activity		
Variable Name	Total (min)	Media	pSocial	Nonmedia
Screen	7101	24	46	30
Audio	8631	28	23	47
Voice	927	42	11	47
Text	5373	45	21	32
Game	1289	57	34	9
pRead	3543	34	5	61
eRead	2663	70	9	21
eWork	2728	50	17	33
eShop	364	62	27	11
eSocial	4089	73	10	16
pSocial	26891	31	–	69
Class	6537	21	35	44
pWork	6866	52	8	40
Employment	1776	5	82	14
Care	1759	22	51	23
Chores	2519	27	48	25
pShop	1484	10	57	33
Foot	2945	25	55	20
Car	1392	29	55	9
Bus	763	40	40	18
Exercise	2361	21	61	18
Wait	2323	24	48	27
Personal	2192	54	29	16
Eat	9443	30	58	12
Sleep	609	47	7	46
Overall				
Media	36708	42	23	35
pSocial	26891	31	–	69
Nonmedia	42969	30	43	26
Total	106568	34	25	40

#### Tasking indices

**Figure 1:**
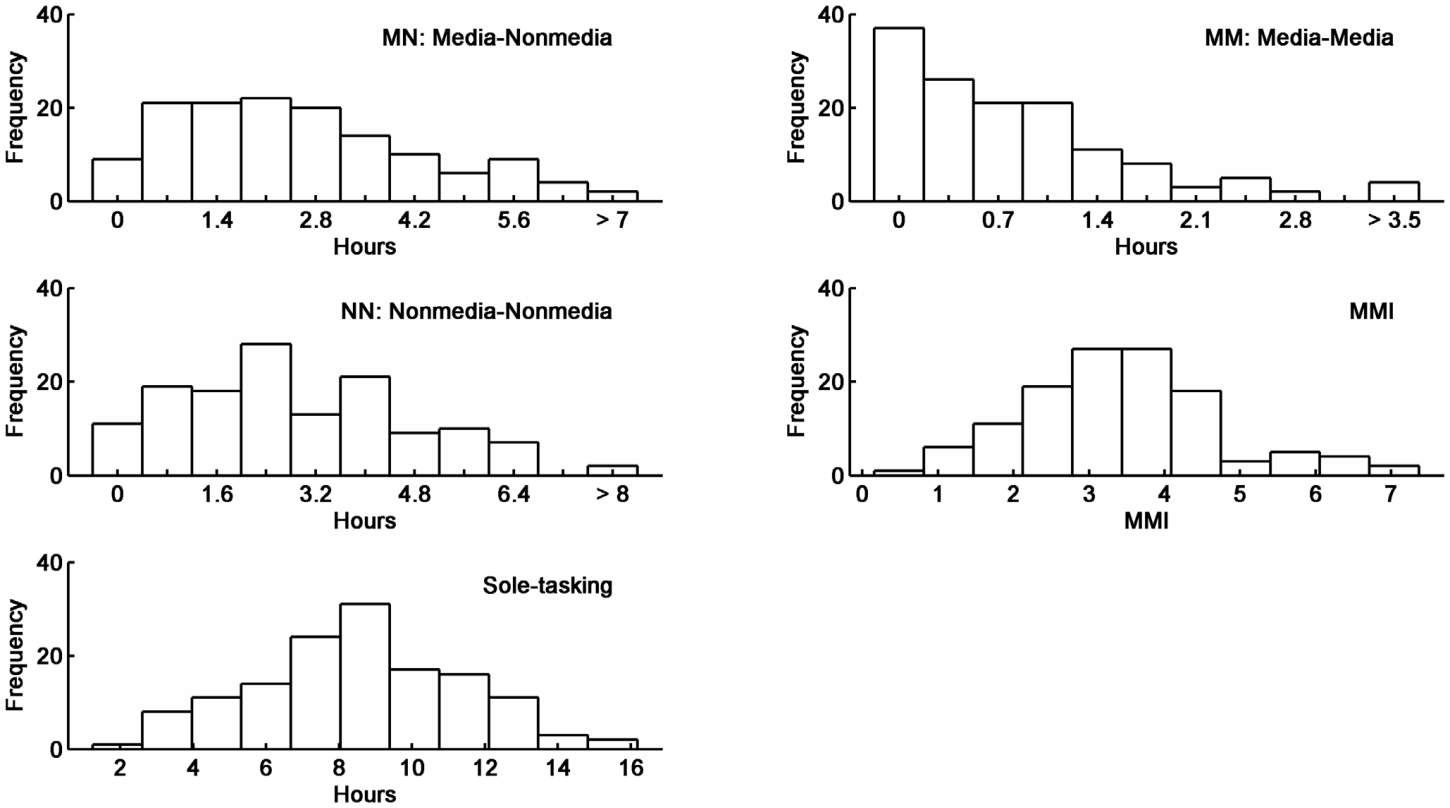
Distributions of tasking indices.

From the SPD, T_MM_, T_MN_, T_NN_, and T_ST_ were obtained for each participant to respectively index the extent of MMM, MNM, NNM and ST. T_MM_ was the total time (in hours) that involved pairs of media activities. T_MN_ was the total time that involved pairs of activities one of which involved one of the media and the other did not. T_NN_ was the total time that involved pairs of nonmedia activities. T_ST_ was the total time that involved solus activities excluding sleep/nap (i.e., sole-tasking). Figure 1 displays the distribution of each of these indices as well as MMI; Table 5 presents their mean and SD and bivariate correlations. Nonparametric (i.e., Spearman's *rho*) correlations were computed because the indices were not normally distributed (Figure 1). The correlations were controlled for age and gender although the pattern of results was identical to that without such controls. [A program for computing nonparametric partial correlations in R can be downloaded from http://www.yilab.gatech.edu/pcor.html, Accessed 2013 Apr 17.] Statistical significance was evaluated at an alpha level of .05/10 (i.e., .005) because 10 correlations were computed. There was a significant correlation between T_MM_ and T_MN_, indicating greater extent of MMM was associated with greater extent of MNM. T_ST_ was significantly, negatively correlated with T_MM_, T_MN_, and T_NN_, indicating greater extent of sole-tasking was associated with smaller extent of multitasking. Notably the correlation between T_MM_ and MMI was non-significant, *p* = .037>.005. This apparent anomaly will be addressed in the Discussion.

**Table 5:** Mean and SE for Tasking Indices and their Spearman Correlations (and p Values) after Controlling for Age and Gender.

Index	Mean	SD	T_MM_	T_MN_	T_NN_	T_ST_
Estimate based on the previous day						
T_MM_	0.92	1.32	–			
T_MN_	2.55	1.75	.24 (.004)*	–		
T_NN_	2.89	1.92	−.04 (.649)	.15 (.094)	–	
T_ST_	8.52	2.85	−.26 (.002)**	−.48 (8E-10)***	−.60 (2E-17)***	
MMI	3.41	1.28	.19 (.037)	.13 (.169)	.05 (.598)	−.12 (.181)

*N* = 138 except where MMI is concerned (*N* = 123).

MM  =  Media-Media; MN  =  Media-Nonmedia; NN  =  Nonmedia-Nonmedia; ST  =  Sole-tasking; MMI  =  Media Multitasking Index.

* *p*<.05/10 = .005; ** *p*<.01/10 = .001; *** *p*<.001/10 = 1E-4.

### Multiple Regressions

#### Data Reduction

Descriptive statistics for the psychosocial variables are presented in Table 6. By definition, some psychosocial variables derived from the questionnaires and languages analyses appear to measure similar construct (e.g., the two well-being related scales). Thus, the factorability of the 13 psychosocial variables was explored first using principal components factor analyses with varimax rotation. [Different rotation methods yielded identical factor structure.] The Mutitasking Preference Inventory (MPI) and emotional positivity variable were excluded from further factor analyses because a low Kaiser-Meyer-Olkin value was obtained for the MPI (.339) and a low communality was obtained for the emotional positivity variable (.388). From the remaining 11 variables, three factors (i.e., well-being, sociability, and impulsivity) were extracted and they respectively accounted for 24, 20, and 15% of the total variance. The factor structure is displayed in Table 7. Using.5 as the cut-off loading value, the well-being factor consisted of measures from the Ryff's Scales of Psychological Well-being, Agreeableness Scale, Warwick-Edinburgh Mental Well-being Scale, and Attention Control Questionnaire; the sociability factor consisted of total word count, making distinctions, psychological distancing, and social engagement; the impulsivity factor consisted of Barratt Impulsiveness Scale, Sensation Seeking Scale, and Extraversion Scale. For each factor, a composite score was computed using the Horst method [35]. Prior to the computation, psychological distancing scores were reversed because its factor loading was negative.

**Table 6:** Cronbach's Alpha, Mean and SE for Various Measures.

Measure	Alpha	Scale	Mean	SE	*n*	
Multitasking Preference Inventory	.88	1–5	2.61	0.06	117	
The Ryff Scales of Psychological Well-Being	.93	1–6	4.16	0.09	117	
Warwick-Edinburgh Mental Well-being Scale	.88	1–5	3.56	0.05	117	
Barratt Impulsiveness Scale, Version 11	.83	1–4	2.14	0.03	117	
Attention Control Questionnaire	.82	1–4	2.41	0.03	117	
Sensation Seeking Scale	.74	1–5	3.50	0.07	117	
Agreeableness Scale	.78	1–5	3.79	0.06	117	
Extraversion Scale	.86	1–5	3.30	0.07	117	
Language analyses (LIWC2007)^a^						
Total word count	–	–	889.15	46.45		134
Emotional positivity	–	–	1.75	0.12		134
Psychological distancing	–	–	0.02	0.05		134
Making distinctions	–	–	−0.03	0.04		134
Social engagement	–	–	−0.03	0.07		134

a Four (out of 138) participants' text files were corrupted.

**Table 7:** Factor Loadings and Communalities Based on a Principle Components Analysis with Varimax Rotation (*n* = 113).

Measures	Factor			Communality
	Wellbeing	Sociability	Impulsivity	
The Ryff Scales of Psychological Well-Being	**.84**	.15	.13	.75
Agreeableness Scale	**.67**	.06	.00	.45
Warwick-Edinburgh Mental Well-being Scale	**.65**	.02	.10	.44
Attention Control Questionnaire	**.65**	−.18	−.29	.54
Total word count	.18	**.79**	−.07	.66
Making distinctions	−.11	**.79**	−.01	.63
Psychological distancing	.09	−**.77**	−.03	.60
Social engagement	.15	**.67**	.14	.48
Barratt Impulsiveness Scale, Version 11	−.38	−.02	**.79**	.77
Sensation Seeking Scale	.13	.03	**.75**	.58
Extraversion Scale	.47	.08	**.60**	.59

#### Stepwise Multiple Regressions

In the absence of relevant literature or theory to guide hypothesis testing, stepwise multiple regression analyses were conducted to explore the relationships between respective psychosocial measures and tasking indices and MMI. That is, the criterion (dependent) variable in an analysis was a psychosocial measure, while the predictor (independent) variables were T_MM_, T_MN_, T_NN_, T_ST_, and MMI. In each analysis, the age and gender variables were entered first as control variables; then a stepwise method was used to select, if any, the best predictor or best combination of predictors using *p*< = .05 and *p* > = .10 respectively as entering and removing criteria. All variables (except gender) were centred. Normal probability plots were used to examine normality of residuals. Statistical significance of selected predictors was evaluated at an alpha level of .05/5 (i.e., .01) because five predictors were entered in each stepwise regression [36]. The results showed two marginally significant relationships – T_MN_ was positively associated with the emotional positivity factor (β = .206, *p* = .026) and the impulsivity factor (β = .196, *p* = .035). However, no relationship reached the .01 significance level.

## Discussion

The present study developed a new instrument – the Survey of the Previous Day (SPD) – and four tasking indices. Relative to existing measures of MMM such as the Media Use Diaries (MUD) and Media Use Questionnaire (MUQ), the SPD is more inclusive (i.e., an activity is counted no matter how briefly it is engaged in) and more objective (i.e., with memory aide and operationally defined estimation methods).

### Media Multitasking

The histogram of MMI was clearly different from that of T_MM_ (Figure 1). Twenty (out of 138) participants scored zero for T_MM_ (i.e., they did not engage in MMM), while none scored zero for MMI (minimum  = 0.49). The survey by the Kaiser Family Foundation also showed 15 to 20 percent of American youth did not engage in MMM [7]. Thus, the skewed histogram for T_MM_ was unlikely an anomaly. However, this characteristic was not reflected in the histogram of MMI. In addition to the low similarity in histograms between MMI and T_MM_, the effect sizes for MMI-T_MM_ correlation was small (.19). To probe the poor relationships, estimated total media use time (in hours) obtained from the MUQ was compared with those estimated from the SPD (i.e., total time per day multiplied by 7 days) with respect to comparable activity categories (see Table 8). Although the two sets of estimates were positively correlated, *rho*(867)  = .58, *p*<.001, the estimates obtained from the MUQ were significantly greater than those from SPD in all but one (Game) of the categories. The overall difference amounted to more than 48 hours per week! Although it is highly probable that participants might spend more time on MMM during weekends, the additional time would be highly unlikely to be near 48 hours. This extent of difference was more likely due to the present sample failing to provide reliable responses to the MUQ, consistent with the finding of Greenberg et al. [10] that survey methods (e.g., MUQ) were less accurate than diary method (e.g., SPD). To increase reliability of the MUQ, perhaps provide a work sheet for participants to estimate the number of hours for each activity for each day (or for weekday and weekend) separately before adding them up. Further research is being carried out to understand the small correlation between MMI and T_MM._

**Table 8:** Estimated Total Number of Hours per Week for Comparable Media Activity Categories.

Media Use Questionnaire (A)			Survey of the Previous Day (B)			A–B		
Media	Mean	SE	Variable name	Mean	SE	Mean	SE	99% CI
Television; Computer-based video	18.00	1.05	Screen	12.57	0.95	5.43	1.12	[2.50, 8.36]
Music; Nonmusic audio	16.14	1.30	Audio	8.37	1.00	7.77	1.33	[4.30, 11.25]
Video or computer games	3.33	0.72	Game	1.85	0.40	1.48	0.61	[−0.12, 3.07]
Instant messaging; SMS; Email	19.45	2.62	Text	6.21	0.65	13.25	2.58	[6.50, 19.99]
Telephone and mobile voice calls	5.36	0.65	Voice	2.54	0.35	2.83	0.69	[1.03, 4.63]
Print media	9.24	0.95	pRead	5.46	0.71	3.78	0.85	[1.56, 5.99]
Web surfing; Other computer-based applications	26.21	2.03	eRead; eWork; eShop; eSocial	12.26	1.04	13.95	2.06	[8.56, 19.35]

### Multitasking Preference Inventory (MPI) and Tasking Indices

The MPI scores were poorly correlated with multitasking indices (.02< |*rho*| <.15). Considering the items in the MPI, this finding is not surprising. The MPI concerns projects and assignments at work places (e.g., “I prefer to work on several projects in a day, rather than completing one project and then switching to another.”; “I like to finish one task completely before focusing on anything else.”). They are often externally imposed. The multitasking indices were estimated from ‘ordinary’ daily life, in which multitasking is often self-indulgent. Thus, multitasking behaviours in daily life do not necessarily mirror multitasking preference at work places.

### Correlations between Tasking Indices and Psychosocial Factors

The regression analyses did not reveal significant correlations between tasking indices and psychosocial factors. The results suggest that the extent of multitasking (MMM, MNM, or NNM) and sole-tasking were not associated with well-being, emotional positivity, sociability, or impulsivity for the present sample. Consistent with Ophir et al. [9] which showed no correlation between MMI and agreeableness or extraversion, the present study showed no significant correlation between MMM and well-being factor (which includes agreeableness) and impulsivity factor (which includes extraversion).

### Miscellaneous Observations

Inspecting Table 3, it is apparent that the multitasking pattern of same activity (e.g., read, work) varied with whether the activity was conducted electronically or not. For clarity, Table 9 presents the proportion time as a function of the 25 activities for each of the relevant activities. Clearly, when the same activity was carried out electronically, it was more likely to be paired with other electronic activities; however, it was far more likely to be paired with non-electronic activities when it was not carried out electronically.

**Table 9:** Multitasking Pattern of Same Activity Varied with Whether the Activity was Conducted Electronically.

	Social		Read		Work		Shop	
	eSocial	pSocial	eRead	pRead	eWork	pWork	eShop	pShop
Electronic activity								
Screen	.10	.12	.03		.03	.02	.03	.02
Audio	.21	.07	.10	.07	.05	.07	.21	.03
Voice								
Text	.19	.04	.10	.02	.02	.02	.04	.04
Game	.03	.02	.02					
eRead	.14		–	.06	.19	.05	.05	
eWork	.02	.02	.15	.16	–	.09		
eShop	.02						–	
eSocial	–	.02	.21		.03	.02	.28	
*Total*	.*72*	.*29*	.*41*	.*32*	.*30*	.*26*	.*34*	.*09*
Non-electronic activity								
pRead			.08	–	.19	.25		
pSocial	.10	–	.09	.05	.16	.08	.27	.57
Class	.02	.08	.03	.07	.07	.37		
pWork	.03	.02	.13	.48	.22	–		
Employment		.05					.02	
Care		.03		.01				.04
Chores		.04						
pShop		.03						–
Foot		.06						.09
Car		.03						
Bus								
Exercise		.05						
Wait		.04						.12
Personal		.02						
Eat	.06	.20	.03	.03	.02		.09	.05
Sleep								
*Total*	.*28*	.*70*	.*38*	.*66*	.*67*	.*73*	.*38*	.*90*

Variable names are defined in Table 1.

Values < = .01 are suppressed.

Descriptions were generally written adequately with respect to language rules except that a few scripts omitted periods at the end of some sentences and some inaccurate uses of punctuations. All words were spelt out except for common British abbreviations (e.g., uni for university). No single instance of text-speak was revealed. Overall, there was no evidence of poorly written communications.

### Limitations

Participants in this study were university students and most of them completed the SPD on a Wednesday. Their schedules and activities would be different from those of other age groups and populations (e.g., high school pupils, office workers, and pilots), to which the current results may not necessarily generalise. Tasking measures were based on the data from a single day. To increase reliability of the tasking measures and to examine whether (and how) they may vary with the day of a week, it would be desirable to invite participants to take the SPD multiple times so that different days in a week would be sampled.

## Conclusions

The Survey of the Previous Day (SPD) is a useful alternative for indexing the extent of MMM. The qualitative data provide memory aide to increase reliability in reporting activities, their timing and duration. Using the SPD, we can minimise omissions due to a coarse-grained record (e.g., report an activity if it had been carried out for at least 15 minutes). The SPD allows objective methods for estimating multitasking time, instead of leaving participants to choose their own methods for estimation (as in MUQ) that are unknown to researchers. Moreover, from the SPD indices for the extent of MMM and other forms of multitasking and sole-tasking can be estimated and a multitasking profile (Table 3) can be created for further inferences and additional insights. The results showed that MMM did not appear to be associated with widespread harmful effects for the present sample.

## Supporting Information

Appendix S1 (DOCX)
