# Supplementary material for: A Null Relationship between Media Multitasking and Well-Being
Source: PLoS One. 2013 May 15;8(5):e64508. doi: 10.1371/journal.pone.0064508 (PMC3655149; doi:10.1371/journal.pone.0064508)
Supplement: Appendix S1 — (DOCX) [file pone.0064508.s001.docx]

**APPENDIX**

Time spent on activity X in the previous day was computed separately for timeslots that involved X only (solus timeslots) and that involved X and one or more other activity (shared timeslots). For solus timeslots, X was credited with the entire 10 minutes of each timeslot.

For each shared timeslot of k activities, concurrency ratings were quantified into fractions of a timeslot as follows -- 'Rarely' = 1/k (i.e., time was equally divided); 'Almost always' = 0.9; the remaining three options were set between 1/k and 0.9 in steps of (0.9 - 1/k)/4. Fractions for each activity were totalled across pairs for that timeslot. Each total was then divided by (k − 1) (a normalized total) because each activity would be paired (k − 1) times. A normalized total with a concurrency rating 'rarely' was counted into solus timeslot; otherwise, it was counted into shared timeslot. These computations are illustrated in the following example.

Suppose a timeslot is shared by three activities A, B, and C (i.e., k = 3). Thus, the incremental step equals (0.9 – 1/3)/4 = 17/120. Hence, the fractions of a timeslot for the five concurrency ratings are respectively 1/3, (1/3 + 17/120), (1/3 + 34/120), (1/3 + 51/120), 9/10 [approximately 0.33, 0.47, 0.62, 0.76, and 0.90]. Suppose the concurrency ratings are as follows: AB pair – 'Rarely'; AC pair – 'Sometimes'; BC pair – 'Frequently'. A and B will each be credited with 0.33/2 solus timeslot. A will be credited with 0.47/2 shared timeslot; B will be credited with 0.76/2 shared timeslot; C will be credited with (0.47 + 0.76)/2 shared timeslot.

The total number of timeslots for activity X was the sum of all solus and shared timeslots concerning X across the 24 hours of the previous day. Multiplying this number by 10 minutes gave the total minutes of activity X. The proportion of multitasking time of X was therefore the total number of shared timeslots of X divided by the total number of timeslots concerning X.
